# Hysteretic Tricolor Electrochromic Systems Based on the Dynamic Redox Properties of Unsymmetrically Substituted Dihydrophenanthrenes and Biphenyl-2,2'-Diyl Dications: Efficient Precursor Synthesis by a Flow Microreactor Method

**DOI:** 10.3390/ma4111906

**Published:** 2011-10-26

**Authors:** Yusuke Ishigaki, Takanori Suzuki, Jun-ichi Nishida, Aiichiro Nagaki, Naofumi Takabayashi, Hidetoshi Kawai, Kenshu Fujiwara, Jun-ichi Yoshida

**Affiliations:** 1 Department of Chemistry, Faculty of Science, Hokkaido University, Sapporo 060-0810, Japan; E-Mails: y_ishigaki@mail.sci.hokudai.ac.jp (Y.I.); jnishida@echem.titech.ac.jp (J.N.); kawaih@rs.tus.ac.jp (H.K.); fjwkn@sci.hokudai.ac.jp (K.F.); 2 Department of Synthetic Chemistry and Biological Chemistry, Graduate School of Engineering, Kyoto University, Kyoto 615-8530, Japan; E-Mails: anagaki@sbchem.kyoto-u.ac.jp (A.N.); tak@mail.sci.hokudai.ac.jp (N.T.); yoshida@sbchem.kyoto-u.ac.jp (J.Y.)

**Keywords:** electrochromism, organic dye, cationic dye, hysteresis, dynamic redox system, reaction integration, flow microreactor

## Abstract

A series of biphenyl-2,2'-diylbis(diarylmethanol)s **3**, which have two kinds of aryl groups at the bay region, were efficiently obtained by integrated flow microreactor synthesis. The diols **3NO/NX** are the precursors of unsymmetric biphenylic dications **2NO/NX**^2+^, which are transformed into the corresponding dihydrophenanthrenes **1NO/NX** via **2NO/NX**^+•^ upon reduction, when they exhibit two-stage color changes. On the other hand, the steady-state concentration of the intermediate **2NO/NX**^+•^ is negligible during the oxidation of **1NO/NX** to **2NO/NX**^2+^, which reflects unique tricolor electrochromicity with a hysteretic pattern of color change [color 1→color 2→color 3→color 1].

## 1. Introduction

Electrochromism [1] is a representative function of organic redox systems, by which electrochemical input is reversibly transduced into UV-Vis spectral output. A vivid color change is a desirable feature of these systems, and thus the stable cationic dye moieties such as triarylmethyliums [2] have often been adopted for this purpose. During the course of our studies on “dynamic redox systems” [3,4] that undergo reversible C–C bond-formation/-breaking upon electron transfer, we found that 9,9,10,10-tetraaryl-9,10-dihydrophenanthrenes (DHP) **1** and biphenyl-2,2'-diylbis(diarylmethylium)s **2**^2+^ constitute a novel series of electrochromic pairs.

**Scheme 1:**
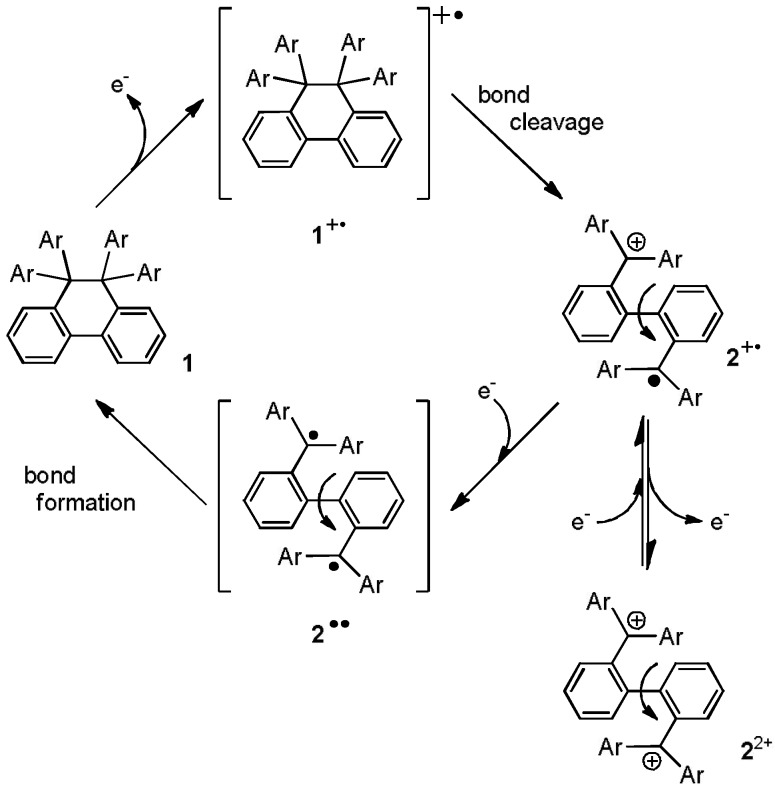
Mechanism of interconversion between **1** and **2**^2+^.

For example, the electron-donating DHPs (**1NN**, **1OO**, **1XX)** show absorptions only in the UV region, whereas the corresponding dications exhibit characteristic vivid colors (blue, red, or yellow) depending on the aryl group [λ_max_/nm (log ε) in MeCN: 661 (4.92), 604 (5.05) for **2NN**^2+^; 539sh (4.72), 514 (4.87) for **2OO**^2+^; 495sh (3.62) 460 (3.79) for **2XX**^2+^] [5,6]. Mechanistic studies have indicated that interconversion proceeds via the biphenyl-2,2'-diyl-type cation radical **2**^+•^ as a common intermediate (Scheme 1) [6,7]. For the *C*2-symmetric derivatives shown above, however, this cation radical intermediate cannot be detected during the oxidation process of **1**, since **2**^+•^ is more easily oxidized than **1** [*E*^ox^(**1**) > *E*^ox^(**2**^+•^)]. The same intermediate **2**^+•^ is also short-lived during the reduction of **2**^2+^, since **2**^+•^ undergoes facile disproportionation [*E*_1_^red^(**2**^2+^)~*E*_2_^red^(**2**^2+^)] into **2**^2+^ and **2**^••^, the latter of which is instantaneously converted to **1** by intramolecular C–C bond-formation. In this way, two-color switching [color 1 (**1**) <=> color 2 (**2**^2+^)] is induced upon the application of electrochemical input to redox pairs with *C*2-symmetry.

We envisaged that the proper molecular design could incorporate the intermediate **2**^+•^ in a novel tricolor electrochromism: disproportionation of **2**^+•^ to **2**^2+^ and **2**^••^ could be suppressed by attaching two kinds of aryl groups with different electron-donating properties. In this report we describe the preparation and novel electrochromic behavior of unsymmetrically substituted pairs such as **1NO**/**2NO**^2+^ or **1NX**/**2NX**^2+^. Flow microreactor synthesis [8,9,10,11,12,13,14,15,16,17,18,19] was very effective for sequentially attaching two different diarylmethyl units to the biphenyl skeleton, whereas the conventional macro batch method gave the desired products in low yields (ca. 10%). The color changes show hysteresis, in that there is a difference between oxidation [color 1 (**1**)→color 2 (**2**^2+^)] and reduction [color 2 (**2**^2+^)→color 3 (**2**^+•^)→color 1 (**1**)].

## 2. Results and Discussion

### 2.1. Preparation of Unsymmetrically Substituted Diol-Precursors

**Scheme 2:**
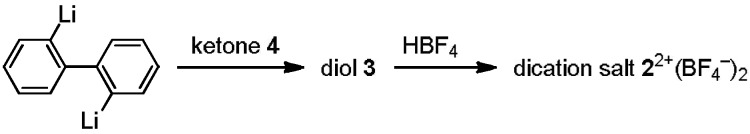
Preparation of precursor diols **3** under the macro batch conditions.

Biphenyl-2,2'-diylbis(diarylmethanol)s **3** are the precursors of dicationic dyes **2**^2+^ [5]. Diols with *C*2-symmetry were readily obtained by reacting diarylketone **4** and 2,2'-dilithiobiphenyl, the latter of which was generated *in situ* from 2,2'-dihalobiphenyl and 2 equiv. of BuLi at −78 °C (Scheme 2) [20]. To prepare unsymmetric diols **3NO** or **3NX** with two different diarylmethane units, we first used a mixture of two diarylketones (**4N-4O**, **4N-4X)** as an electrophile. Though the desired unsymmetric diols were obtained as a mixture containing two symmetric diols and other byproducts, tedious chromatographic separation only afforded pure **3NO** or **3NX** in respective yield of 12% and 9% [6]. Attempts to improve the yield were unfruitful even when dihalobiphenyl was treated sequentially with BuLi (1 equiv.), diarylketone (1 equiv.), BuLi (another 1 equiv.), and another diarylketone (1 equiv.). Again, a mixture of three diols was obtained, since selective monolithiation is difficult under the conventional macro batch conditions even if 1 equiv. of BuLi is used [21].

**Figure 1:**
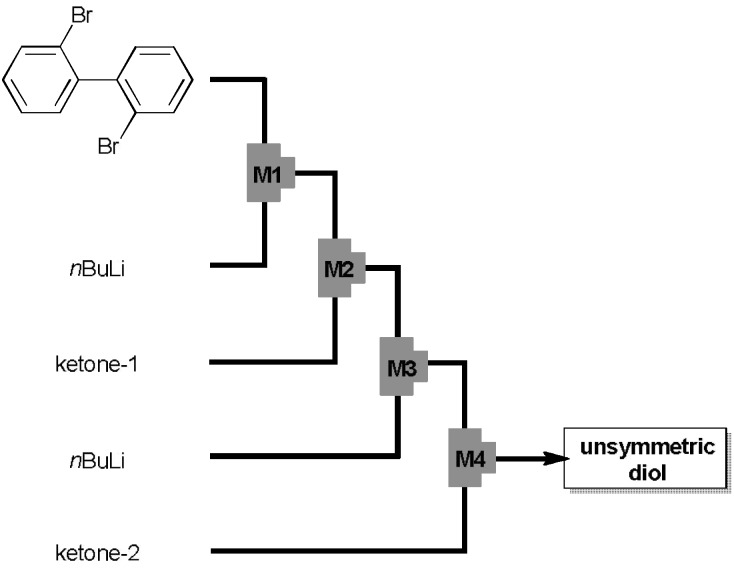
An integrated flow microreactor system for the sequential introduction of two different diarylmethanol units at the 2,2'-position of biphenyl.

On the other hand, some of us recently demonstrated that monolithiation of dibromobiphenyl can be successfully conducted under the flow microreactor conditions [22,23]. By taking advantage of this process, we succeeded in the sequential introduction of two diarylmethyl units by reaction integration using flow microreactor synthesis [24,25,26] (Figure 1) thanks to fast micromixing and precise temperature control. The best result was obtained when 0.1 M 2,2'-dibromobiphenyl in THF (flow rate 6.0 mL/min) was reacted with 0.5 M BuLi in hexane (1.2 mL/min) for 0.06 s at 24 °C to generate 2-lithio-2'-bromobiphenyl, which was sequentially reacted with ketone **4O** (0.2 M in THF, 3.0 mL/min), BuLi (0.5 M in hexane, 1.44 mL/min), and another ketone **4N** (0.1 M in THF, 7.2 mL/min). This sequence of reactions proceeded in a short period of 15.5 s, and, unsymmetric diol **3NO** with 4-dimethylaminophenyl and 4-methoxyphenyl groups was generated in high NMR yield of 92% and isolated in 73% yield after chromatography. Under similar conditions, **3NX** with 4-dimethylaminophenyl and xanthenyl groups was prepared in 81% NMR yield and isolated in 61% yield.

**Scheme 3:**
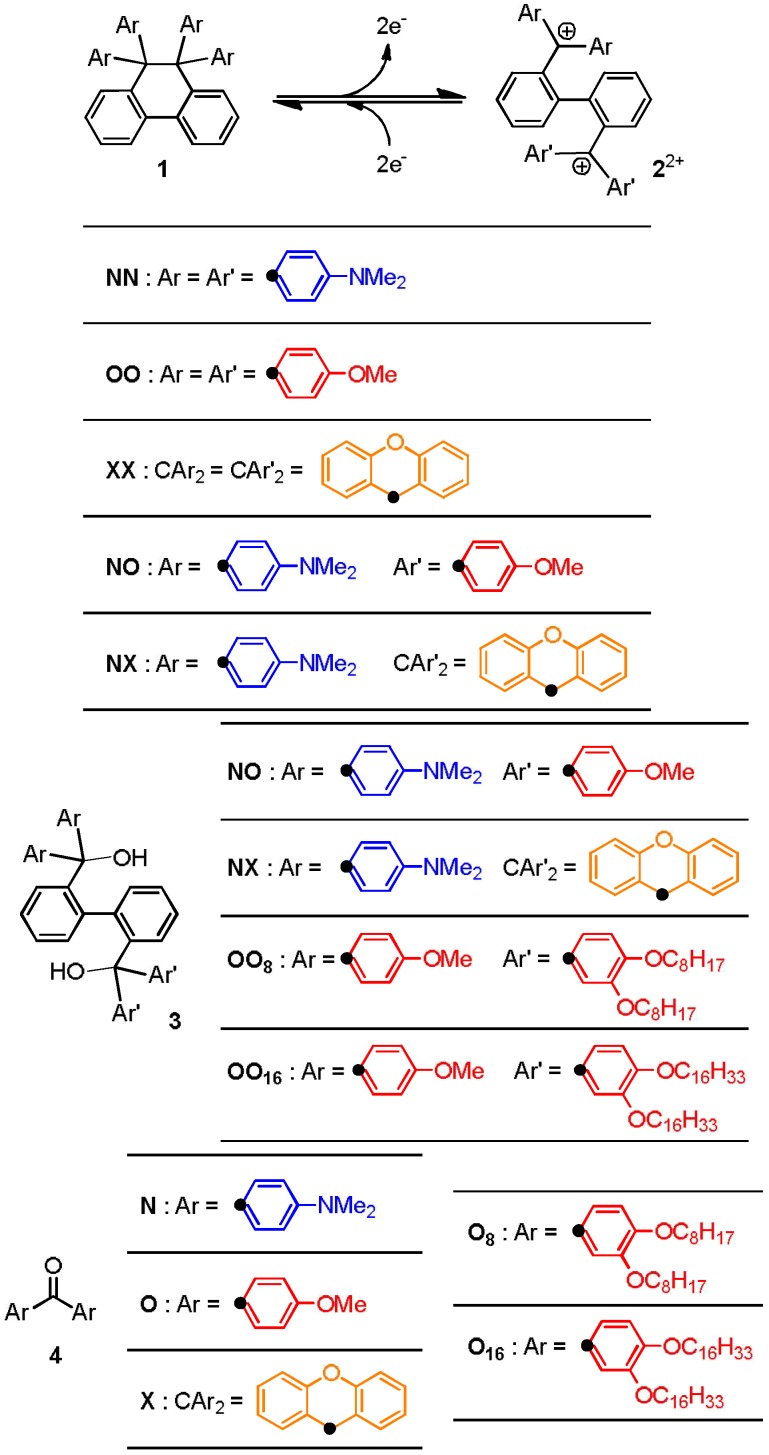
Formula of compounds

In both **3NO** and **3NX,** the two diarylmethyl units differ significantly in terms of their electron-donating properties, so that the combination would be suitable for suppressing the disproportionation of the cation radical intermediate (**2NO**^+•^ and **2NX**^+•^) during the electrochemical interconversion of **1** and **2**^2+^. In addition to its value in preparing the precursors of tricolor electrochromic systems shown above, the flow microreactor method can also produce another group of materials by adopting other bulky diarylketones with long alkoxy chains as an electrophile [e.g., 3,3',4,4'-tetrakis(octyloxy)- or tetrakis(hexadecyloxy)benzophenones, **4O_8_** or **4O_16_**]. Unsymmetrically substituted diols **3OO_8_** and **3OO_16_** were obtained in good isolated yields of 68% and 62%, respectively, and could be used to generate unique dicationic dyes that are soluble in a hydrocarbon solvent [7]. The results shown above clearly demonstrate that the flow microreactor system is very effective for the sequential introduction of two bulky substituents at the bay region of the biphenyl skeleton.

### 2.2. Preparation and Two-Electron Interconversion of Unsymmetrically Substituted Redox Pairs

When diols **3NO** and **3NX** were treated with HBF_4_-(EtCO)_2_O, deeply colored dications were isolated as stable BF_4_ salts in respective yields of 91 and 93%. In the UV-Vis spectrum of **2NO**^2+^(BF_4_^−^)_2_ [λ_max_/nm (logε) in MeCN: 632 (4.93), 519 (4.72)], both of the strong absorptions that are characteristic of two different triarylmethyliums are present. Thus, the deep purple color of **2NO**^2+^(BF_4_^−^)_2_ solution is due to the simple combination of blue and red chromophores found in **2NN**^2+^ and **2OO**^2+^. Similarly, the green color of **2NX**^2+^(BF_4_^−^)_2_ [632 (4.92), 488 sh (3.76), 425 (4.22)] can be accounted for by the presence of the blue and yellow dye units within the molecule.

These dications were cleanly converted into colorless DHPs, **1NO** and **1NX**, upon reduction with SmI_2_ in THF in respective yields of 97 and 78%. The resulting DHPs were reoxidized to **2NO**^2+^ and **2NX**^2+^ by treatment with 2 equiv. of (4-BrC_6_H_4_)_3_N^+•^ SbCl_6_^−^ in CH_2_Cl_2_, which were in turn isolated as deeply colored SbCl_6_^−^ salts in respective yields of 82 and 79%. This high-yield interconversion indicates that **1NO**/**2NO**^2+^ and **1NX**/**2NX**^2+^ constitute a “reversible” redox pair although C–C bond-formation/-breaking is accompanied by electron transfer (“dynamic” redox behavior) [3,4].

**Figure 2:**
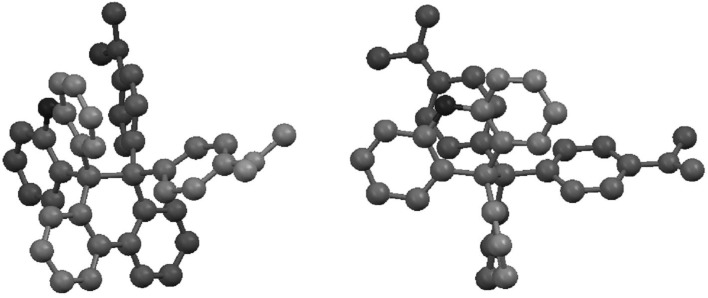
Molecular structure of **1NX** determined by X-ray analysis: (**left**) top view; (**right**) side view. The DHP skeleton adopts a helical geometry, and all of the molecules in a single crystal are homochiral in terms of helicity.

**Scheme 4:**
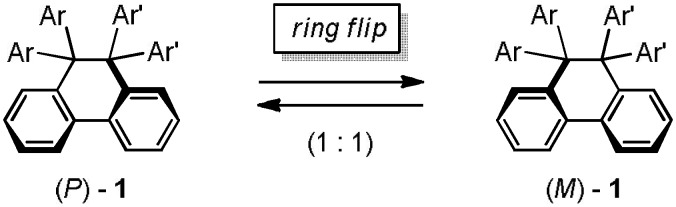
Interconversion of the enantiomers of helical DHPs **1** by ring flip.

According to an X-ray analysis of **1NX** (Figure 2), the C9–C10 bond of the DHP unit [1.643(6) Å] is longer than standard (1.54 Å) [27], which is due to the “front strain” [28] among the aryl groups at C9 and C10, as in the case of other polyarylated cyclic compounds [29,30,31,32]. The DHP skeleton adopts a half-chair conformation with a dihedral angle of 16.2° for two benzene nuclei, which endows the molecule with an asymmetric element of helicity (*P*/*M*). In a single crystal of **1NX** (space group: *P*2_1_2_1_2_1_), all of the molecules adopt the same helicity, which shows that spontaneous resolution occurs. Based on the result of a VT-NMR experiment, however, the spectrum indicates *C*1-symmetry only at low temperature (*N*-methyl protons: 2.95 and 2.80 ppm in CDCl_3_), and *C*s symmetry is attained at room temperature (*T*c = −40 °C). *P*/*M*-**1NX** readily undergoes racemization due to facile ring-flip in solution (ΔG^‡^ = 11.4 kcal mol^−1^ at −40 °C) (Scheme 4), while this process is prohibited in the crystal.

### 2.3. Hysteretic Redox Behavior of Unsymmetrically Substituted Redox Pairs

**Figure 3:**
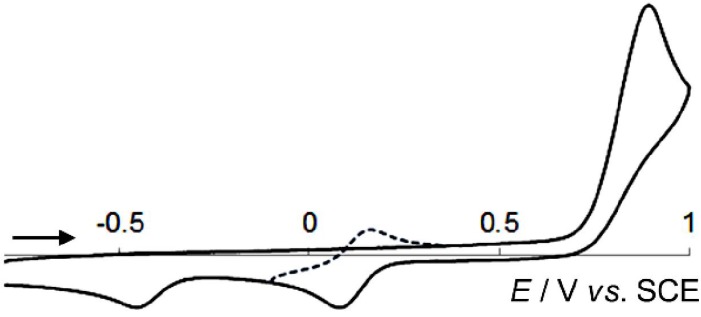
Cyclic voltammogram of DHP **1NO** (10^−3^ M) in CH_2_Cl_2_ (*E*/V *vs*. SCE 0.1 M Bu_4_NBF_4_, Pt electrode, scan rate 500 mV s^−1^). The reduction peaks are absent when the voltammogram is first scanned cathodically. As shown by the dotted line, the first reduction wave at +0.07 V is reversible when the scanning is reversed at −0.10 V.

The cyclic voltammograms of *C*2-symmetric DHPs are similar to each other, with an irreversible 2e-oxidation peak at +0.77 (**1NN**), +1.47 (**1OO**) or +1.42 (**1XX**) V *vs*. SCE in CH_2_Cl_2_, respectively [5,6]. The return wave is largely shifted to the cathode, and was assigned to the 2e-reduction peak of **2NN**^2+^ (−0.45 V), **2OO**^2+^ (+0.18 V), or **2XX**^2+^ (+0.50 V), respectively (Table 1). Similarly, the present unsymmetric DHPs **1NO** and **1NX** undergo irreversible 2e-oxidation (Figure 3). Their oxidation potentials are close to that of **1NN**, indicating that the HOMO level of **1NO** or **1NX** is close to that of **1NN** due to the dimethylaminophenyl groups with strong electron-donating properties. The irreversibility of the oxidation wave suggests that the as-generated cation radical, **1NO**^+•^ or **1NX**^+•^, readily isomerizes to **2NO**^+•^ or **2NX**^+•^ by C9–C10 bond fission. In the return cycle of the voltammogram of **1NO** or **1NX**, two cathodic peaks were seen: such behavior is quite different from that of *C*2-symmetric compounds.

**Table 1:** Redox potentials of **1** and **2**^2+^ in CH_2_Cl_2_^a^.

compd.	*E*^ox^ (1)	*E*_1_^red^ (2^2+^)	*E*_2_^red^ (2^+•^)
**NO**	+0.83^b,c^	+0.10^c^	−0.45^c^
**NX**	+0.76^b,c^	+0.24^c^	−0.19^c^
**NN**	+0.74^b,c^	−0.42^b,c^	
**OO**	+1.44^b,c^	+0.21^b,c^	
**XX**	+1.39^b,c^	+0.53^b,c^	

^a^ *E*/V *vs*. SCE, 0.1 M Bu_4_NBF_4_, Pt electrode, scan rate 100 mV/s; ^b^ Two-electron process; ^c^ Irreversible wave, values are calculated. as *E*^ox^ = *E*_peak_ − 0.03 and *E*^red^ = *E*_peak_ + 0.03.

Independent measurements of **2NO**^2+^ and **2NX**^2+^ confirmed that the two cathodic peaks are due to two-stage 1e-reduction processes of the unsymmetric dications. The first process is completely reversible, and corresponds to the reduction of bis(4-methoxyphenyl)methylium in **2NO**^2+^ or the xanthenylium moiety in **2NX**^2+^. Furthermore, after scanning of the irreversible second 1e-reduction wave of **2NO**^2+^ and **2NX**^2+^, the anodic peak due to the oxidation of **1NO** or **1NX** appears in the far anodic region of the voltammograms. Such redox properties can only be accounted for by assuming the reaction mechanism shown in Scheme 1, where the elongated C9–C10 bond in DHP is cleaved after just 1e oxidation of **1NO** to **1NO**^+•^[33] whereas two-fold 1e-reduction of **2NO**^2+^ to **2NO**^••^ is necessary before the ring closure. **2NO**^+•^ produced from **1NO**^+•^ is more easily oxidized than **1NO** [*E*^ox^(**1NO**) = +0.83 V; *E*^ox^(**2NO**^+•^) = *E*_1_^red^(**2NO**^2+^) = +0.10 V], and thus the steady-state concentration of **2NO**^+•^ is negligible during the electrochemical oxidation of **1NO**, although the same specimen is a long-lived intermediate in the reduction of **2NO**^2+^ due to suppression of disproportionation. The same is true for another series of compounds, **1NX**^+•^/**1NX**^+•^/**2NX**^+•^/**2NX**^2+^ [*E*^ox^(**1NX**) = +0.76 V; *E*^ox^(**2NX**^+•^) = *E*_1_^red^(**2NX**^2+^) = +0.24 V; *E*_2_^red^(**2NX**^2+^) = −0.19 V]. Thanks to the hysteretic interconversion in redox reactions, unique tricolor electrochromic systems could be constructed using the present unsymmetric derivatives, as shown below.

### 2.4. Hysteretic Tricolor Electrochromicity of Unsymmetrically Substituted Redox Pairs

**Figure 4:**
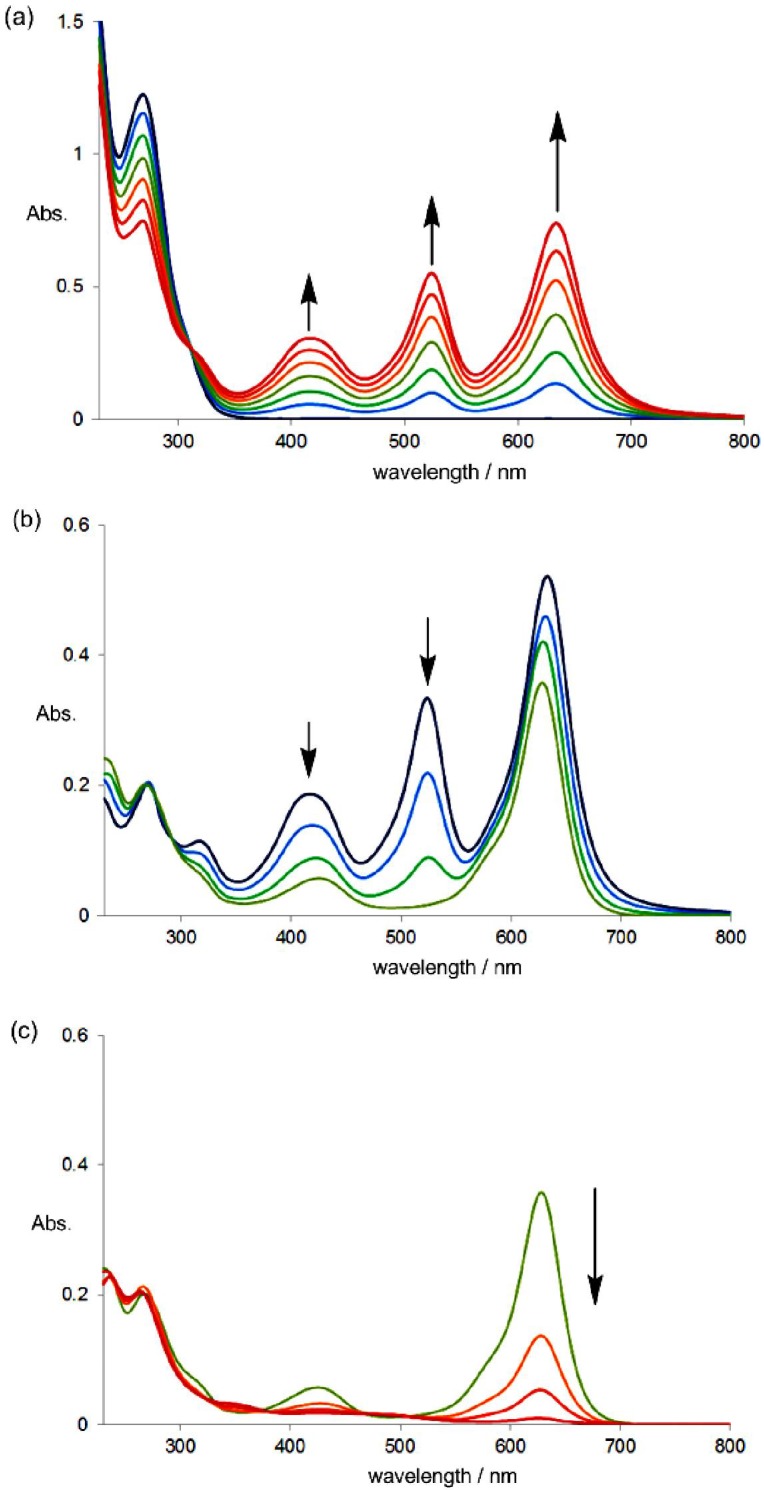
Changes in the UV–VIS spectra of (**a**) **1NO** (3.5 mL; 3.0 × 10^−5^ M in CH_2_Cl_2_ containing 0.05 M Bu_4_NBF_4_) upon electrochemical oxidation (10 μA) at 5-min intervals, and **2NO**^2+^ (3.5 mL soln; 6.2 × 10^−6^ M in CH_2_Cl_2_ containing 0.05 M Bu_4_NBF_4_) upon electrochemical reduction (40 μA): (**b**) stage 1, at 2-min intervals; (**c**) stage 2, at 8-min intervals.

**Scheme 5:**
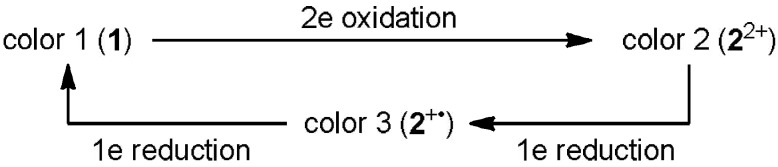
Hysteretic color changes in the novel tricolor chromic systems.

Upon the electrochemical oxidation of colorless **1NO** in CH_2_Cl_2_, both the blue and red chromophores grow simultaneously to develop a violet color for **2NO**^2+^ (Figure 4(a), isosbestic point at 310 nm). On the other hand, the red chromophore predominantly disappears in the first stage of the electrochemical reduction of **2NO**^2+^ (Figure 4(b), 295 nm), and the blue cation radical **2NO**^+•^ is then converted to colorless **1NO** (Figure 4(c), 290 nm) even under constant-current electrolytic conditions (Scheme 5). A similar behavior, but with different colors, was observed for the xanthene derivative. Thus, colorless donor **1NX** was transformed directly into green **2NX**^2+^ (isosbestic point: 309 nm), whereas reduction is a two-stage process; *i.e.*, green **2NX**^2+^ changes to blue **2NX**^+•^ (248, 270, 300 nm) and blue **2NX**^+•^ changes to colorless **1NX** (296 nm), which shows the generality of the unique pattern of the color change.

## 3. Conclusions

Compared with the successful examples based on the polymeric materials [34,35,36,37,38,39,40,41,42,43,44,45,46], tricolor electrochromicity based on discrete molecules is rare, for which only a limited number of examples have been reported [47,48,49,50,51]. Even among successful examples, a hysteretic pattern for the color change, where there is a difference between oxidation [color 1→color 2] and reduction [color 2→color 3→color 1], is quite unique for the present systems. This novel chromicity is attained by the incorporation of biphenyldiyl-type cation radical **2**^+•^ as an additional component to DHP **1** and dication **2**^2+^. The key to extending the lifetime of **2**^+•^ is the different electron-donating properties of two dye chromophores, whose precursors were prepared very efficiently using the flow microreactor method.

**Scheme 6:**
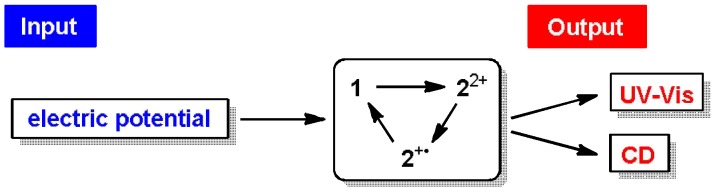
Multi-output response based on the novel tricolor chromic systems.

**Scheme 7:**
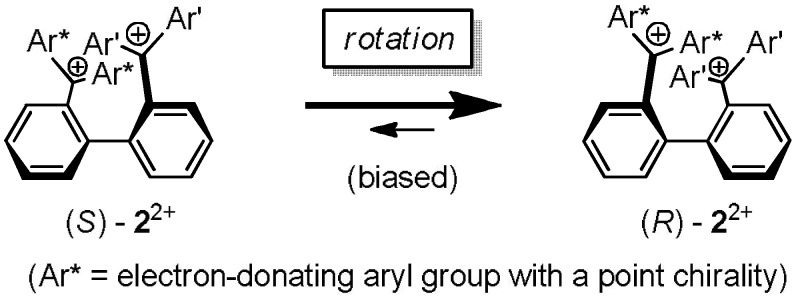
Interconversion of the enantiomers of axially chiral dications **2**^2+^ by rotation to give a diasteromerically biased mixture due to the transfer of point chirality to axial chirality.

We are now developing a new series of tricolor chromic system, which also afford chiroptical properties (e.g., circular dichroism (CD)) as an additional signal, to construct the multi-output response systems (Scheme 6) [3,4,7,52,53,54]. While the triarylmethyliums attached with chiral substituents only exhibit very small ellipticity to be used as an output signal (|Δε| < 1.5), huge enhancement (|Δε| > 100) could be realized by intramolecular transfer of the point chirality to the axial chirality in the biphenyl-diyl dications **2**^2+^ (Scheme 7) [54], in which the two triarylmethylium units are suitably arranged for effective exciton coupling [52,53]. Studies along this vein are now in progress and the results will be reported in due course.

## 4. Experimental Section

### 4.1. Preparation of 9,9-Bis(4-Dimethylaminophenyl)-10,10-Bis(4-Methoxyphenyl)-9,10-Dihydrophenanthrene **1NO**

To a suspension of dication salt of **2NO**^2+^(BF_4_^−^)_2_ (104 mg, 0.129 mmol) in THF (10 mL) was added triethylamine (3 mL) followed by SmI_2_ (0.1 mol dm^−3^ in THF, 5.0 mL, 0.50 mmol) over 10 min at rt. The violet suspension gradually turned to ocher, and then the blue color of SmI_2_ remained persistent during the addition. After stirring for 1 h and removal of THF and amine, the residue was suspended in water and extracted with benzene. The organic layer was washed with water and brine, and dried over K_2_CO_3_. Evaporation of solvent followed by chromatographic separation (Al_2_O_3_, benzene) gave DHP **1NO** as a colorless solid (79 mg, y. 97%).

Mp 284.5–285.5 °C (decomp.); ^1^H NMR (400 MHz, CDCl_3_, 0 °C) *δ*/ppm 7.65–7.71 (2H, m), 7.18–7.25 (2H, m), 7.08–7.18 (4H, m), 6.80–7.06 (8H, m), 6.43–6.55 (4H, AA’XX’), 6.26–6.38 (4H, AA’XX’), 3.71 (6H, s), 2.85 (12H, s); IR (KBr) 2948, 2832, 1610, 1510, 1444, 1354, 1292, 1252, 1186, 1036, 950, 806, 746, 580 cm^−1^; FD-MS *m/z* 630 (M^+^, BP); Anal. Calcd. for C_44_H_42_N_2_O_2_: C, 83.78; H, 6.71; N, 4.44. Found: C, 83.72; H, 6.88; N, 4.48. 

### 4.2. Preparation of Spiro[10,10-Bis(4-Dimethylaminophenyl)-9,10-Dihydrophenanthrene-9,9'-[9H]Xanthene] **1NX**

To a suspension of **2NX**^2+^(BF_4_^−^)_2_ (30 mg, 39.6 μmol) in THF (10 mL) was added SmI_2_ (0.1 mol dm^−3^ in THF, 1.7 mL, 0.17 mmol) over 5 min at rt. The deep green suspension gradually turned to colorless during the addition. After stirring for 30 min and evaporation of THF, the residue was suspended in water and extracted with CH_2_Cl_2_. The organic layer was washed with water and brine, and dried over Na_2_SO_4_. Evaporation of solvent followed by separation by preparative TLC (SiO_2_, hexane/AcOEt = 4) gave DHP **1NX** as a colorless solid (18 mg, y. 78%). Single crystalline specimen of **1NX** was obtained by recrystallization from ether.

Mp 294–295 °C (decomp); ^1^H NMR (400 MHz, CDCl_3_, 24 °C) *δ*/ppm 8.03 (1H, d, J = 7.8 Hz), 7.90 (1H, d, J = 7.8 Hz), 7.46 (1H, ddd, J = 7.8, 7.8, 1.5 Hz), 7.34 (1H, dd, J = 7.8, 1.5 Hz), 7.22–7.30 (3H, m), 7.13–7.19 (2H, m), 6.88–7.05 (3H, m), 6.63–6.72 (6H, m), 6.61 (2H, dd, J = 7.8, 1.5 Hz), 6.24 (4H, AA’XX’), 2.84 (12H, s); IR (KBr) 2796, 1610, 1518, 1480, 1440, 1354, 1312, 1244, 950, 808, 750 cm^−1^; FD-MS *m/z* 584 (M^+^, BP); Anal. Calcd. for C_42_H_36_N_2_O: C, 86.27; H, 6.21; N, 4.79. Found: C, 86.04; H, 6.41; N, 4.65.

### 4.3. Preparation of Biphenyl-2-yl[Bis(4-Dimethylaminophenyl)Methylium]-2'-yl[Bis(4'-Methoxyphenyl)Methylium] Bis(Tetrafluoroborate) **2NO**^2+^(BF_4_^−^)_2_

To a solution of diol **3NO** (117 mg, 0.176 mmol) in DME (3 mL) was added propionic anhydride (0.6 mL) followed by 42% HBF_4_ (6.70 M, 90 μL, 0.60 mmol), and the mixture was heated at 80 °C for 1 h. By slow cooling to rt, dication salt **2NO**^2+^(BF_4_^−^)_2_ was separated as a deep-violet solid (129 mg, y. 91%), which was filtered, washed with DME and dried in vacuo.

Mp 194–198 °C (decomp.); ^1^H NMR (400 MHz, CD_3_CN, 20 °C) *δ*/ppm 7.00–7.70 (16H, m), 6.92–6.98 (4H, AA’XX’), 6.62–6.73 (4H, AA’XX’), 4.03 (3H, s), 4.02 (3H, s), 3.22 (6H, s), 3.17 (6H, s); IR (KBr) 1580, 1506, 1464, 1372, 1278, 1220, 1160, 1124, 1084, 1062, 938, 912, 838, 722 cm^−1^; FAB-MS *m/z* 630 (M^+^, BP); Anal. Calcd. for C_44_H_42_N_2_O_2_B_2_F_8_+0.5H_2_O: C, 64.96; H, 5.33; N, 3.44. Found: C, 65.06; H, 5.40; N, 3.40. 

### 4.4. Preparation of Biphenyl-2-yl[Bis(4-Dimethylaminophenyl)Methylium]-2'-yl[9-Xanthenylium] Bis(Tetrafluoroborate) **2NX**^2+^(BF_4_^−^)_2_

To a solution of diol **3NX** (70 mg, 11.3 μmol) in DME (3 mL) was added propionic anhydride (0.5 mL) followed by 42% HBF_4_ (50 μL, 33.5 μmol), and the mixture was heated at 80 °C for 1 h. By slow cooling to rt, dication salt **2NX**^2+^(BF_4_^−^)_2_ was separated as a deep-green solid (80 mg, y. 93%), which was filtered, washed with DME, and dried in vacuo.

Mp 219–220 °C (decomp.); ^1^H NMR (400 MHz, CD_3_CN, 50 °C) *δ*/ppm 8.40 (2H, br.), 8.23 (2H, br. d, J = 8.5 Hz), 7.90 (2H, br. d, J = 8.5 Hz), 7.78–7.84 (3H, br. m), 7.46–7.56 (3H, br. m), 7.32 (2H, ddd, J = 7.8, 7.8, 1.0 Hz), 7.26–7.30 (1H, br. m), 6.85 (2H, br. d, J = 7.8 Hz), 6.20–7.20 (7H, br.), 3.30 (6H, br. s), 3.29 (6H, br. s); IR (KBr) 1622, 1584, 1506, 1374, 1174, 1084, 1036, 910, 762, 722 cm^−1^; FAB-MS *m/z* 584 (M^+^, BP); Anal. Calcd. for C_42_H_36_N_2_OB_2_F_8_: C, 66.52; H, 4.78; N, 3.69. Found: C, 66.64; H, 4.79; N, 3.76. 

### 4.5. Oxidation to Biphenyl-2-yl[Bis(4-Dimethylaminophenyl)Methylium]-2'-yl[Bis(4'-Methoxyphenyl)Methylium] Bis(Hexachloroantimonate) **2NO**^2+^(SbCl_6_^−^)_2_

To a solution of DHP **1NO** (10 mg, 15.9 μmol) in CH_2_Cl_2_ (10 mL) was added (4-BrC_6_H_4_)_3_N^+•^SbCl_6_^−^ (26 mg, 31.8 μmol), and mixture was stirred for 20 min at rt. Deep-violet precipitates of **2NO**^2+^(SbCl_6_^−^)_2_ (17 mg, y. 82%) were separated upon dilution with hexane (1 mL), which were filtered, washed with CH_2_Cl_2_ and dried in vacuo.

Mp >310 °C (decomp.); IR (KBr) 1580, 1438, 1372, 1280, 1160, 1004, 938, 912, 832, 722, 578 cm^−1^; Anal. Calcd. for C_44_H_42_N_2_O_2_Sb_2_Cl_12_: C, 40.66; H, 3.26; N, 2.16. Found: C, 40.76; H, 3.29; N, 2.26. 

### 4.6. Oxidation to Biphenyl-2-yl[Bis(4-Dimethylaminophenyl)Methylium]-2'-yl[9-Xanthenylium] Bis(Hexachloroantimonate) **2NX**^2+^(SbCl_6_^−^)_2_

To a solution of DHP **1NX** (5.0 mg, 8.6 μmol) in CH_2_Cl_2_ (2.5 mL) was added (4-BrC_6_H_4_)_3_N^+•^SbCl_6_^−^ (14 mg, 17 μmol), and mixture was stirred for 10 min at rt. Deep-green precipitates of **2NX**^2+^(SbCl_6_^−^)_2_ (8.5 mg, y. 79%) were separated upon dilution with hexane (1 mL), which were filtered, washed with CH_2_Cl_2_ and dried in vacuo.

Mp 203–210 °C (decomp.); IR (KBr) 1620, 1582, 1504, 1476, 1372, 1168, 940, 910, 832, 754, 722 cm^−1^; Anal. Calcd. for C_42_H_36_N_2_OSb_2_Cl_12_: C, 40.17; H, 2.89; N, 2.23. Found: C, 39.90; H, 3.05; N, 2.07. 

### 4.7. Preparation of 2-[Bis(4-Dimethylaminophenyl)Hydroxymethyl]-2'-[Bis(4'-Methoxylphenyl)Hydroxymethyl]Biphenyl **3NO** via Flow Microreactor Method

An integrated flow microreactor system consisting of four T-shaped micromixers (M1, M2, M3, and M4), four microtube reactors (R1, R2, R3, and R4), and five microtube units [P1 (inner diameter *φ* = 1000 μm, length *l* = 100 cm), P2 (*φ* = 1000 μm, *l* = 50 cm), P3 (*φ* = 1000 μm, *l* = 100 cm), P4 (*φ* = 1000 μm, *l* = 50 cm), and P5 (*φ* = 1000 μm, *l* = 100 cm)] was used. The whole flow microreactor system was dipped in a water bath (24 °C). A solution of 2,2'-dibromobiphenyl (0.10 M) in THF (flow rate = 6.00 mL min^−1^) and a solution of BuLi (0.50 M) in hexane (flow rate = 1.20 mL min^−1^) were introduced to M1 (*φ* = 250 μm). The resulting solution was passed through R1 (*φ* = 500 μm, *l* = 3.5 cm) and was mixed with a solution of 4,4'-dimethoxybenzophenone **4O** (0.20 M) in THF (flow rate = 3.00 mL min^−1^) in M2 (*φ* = 500 μm). The resulting solution was passed through R2 (*φ* = 1000 μm, *l* = 50 cm) and was introduced to M3 (*φ* = 500 μm) where the solution was mixed with a solution of BuLi (0.50 M) in hexane (flow rate = 1.44 mL min^−1^). The resulting solution was passed through R3 (*φ* = 1000 μm, *l* = 200 cm) and was introduced to M4 (*φ* = 500 μm) where the solution was mixed with a solution of 4,4'-bis(dimethylamino)benzophenone **4N** (0.10 M) in THF (flow rate = 7.20 mL min^−1^). The resulting solution was passed through R4 (*φ* = 1000 μm, *l* = 200 cm). After a steady state was reached, the product solution was collected for 60 s and was treated with BuLi (1.67 M) in hexane (2.0 mL) to consume excess ketones and quench the reaction with water. 

After diluted with H_2_O, the whole mixture was extracted with Et_2_O. The combined organic layers were washed with water and brine, and dried over anhydrous Na_2_SO_4_. After filtration, solvent was concentrated under reduced pressure. The residue was purified by column chromatography on silica gel (hexane/EtOAc = 3) to give **3NO** (291 mg) as a colorless solid in 73% yield.

Mp 221–222 °C (decomp.); ^1^H NMR (400 MHz, CDCl_3_, 24 °C) *δ*/ppm 7.00–7.10 (9H, m), 6.92–6.96 (2H, br. d, J = 8.8 Hz), 6.74–6.86 (9H, m), 6.61–6.65 (4H, m), 6.19 (1H, dd, J = 8.6, 1.0 Hz), 6.04 (1H, dd, J = 8.6, 1.0 Hz), 5.23 (1H, s), 3.83 (3H, s), 3.78 (3H, s) 3.56 (1H, s), 2.95 (6H, s), 2.91 (6H, s); IR (KBr) 3550sh, 2900, 2832, 1612, 1512, 1466, 1352, 1298, 1250, 1174, 1062, 826, 762, 560 cm^−1^; FD-MS *m/z* (rel intensity) 664 (M^+^, 100), 646 (3); Anal. Calcd. for C_44_H_44_N_2_O_4_: C, 79.49; H, 6.67; N, 4.21. Found: C, 79.37; H, 6.80; N, 4.05. 

### 4.8. Preparation of 2-[Bis(4-Dimethylaminophenyl)Hydroxymethyl]-2'-[Bis(4'-Methoxylphenyl)Hydroxymethyl]Biphenyl **3NO** via Macro Batch Method

To a colorless solution of 2,2'-diiodobiphenyl (692 g, 1.70 mmol) in THF (12 mL) was added dropwise BuLi (1.70 mol dm^−3^ in hexane, 2.30 mL, 3.91 mmol) at −78 °C for 5 min under Ar, and the mixture was stirred for 45 min at this temperature. To the resultant suspension of 2,2'-dilithiobiphenyl was added a mixture of 4,4'-bis(dimethylamino)benzophenone **4N** (406 mg, 1.51 mmol) and 4,4'-dimethoxybenzophenone **4O** (364 mg, 1.50 mmol) in THF (30 mL) for 15 min. After stirring for 4 h, BuLi (1.70 mol dm^−3^ in hexane, 1.00 mL, 1.70 mmol) was added for 3 min to consume excess ketones. The resultant red solution was further stirred for 1 h and quenched by adding water. THF was evaporated, and the residue was extracted with benzene. The organic layer was washed with water and brine, and dried over Na_2_SO_4_. Evaporation of the solvent gave 1.09 g of oily material containing unsymmetric diol **3NO**. Two symmetric diols, **3NN** and **3OO**, were also formed in this reaction. Chromatographic separation on SiO_2_ (hexane/AcOEt = 7/3) followed by crystallization from ether gave **3NO** as colorless crystals (120 mg, y. 12%).

### 4.9. Preparation of 9-[2'-Bis(4-Dimethylaminophenyl)Hydoroxymethylbiphenyl-2-yl]-9-Hydroxyxanthene **3NX** via Flow Microreactor Method

An integrated flow microreactor system consisting of four T-shaped micromixers (M1, M2, M3, and M4), four microtube reactors (R1, R2, R3, and R4), and five microtube units [P1 (inner diameter *φ* = 1000 μm, length *l* = 100 cm), P2 (*φ* = 1000 μm, *l* = 50 cm), P3 (*φ* = 1000 μm, *l* = 100 cm), P4 (*φ* = 1000 μm, *l* = 50 cm), and P5 (*φ* = 1000 μm, *l* = 100 cm)] was used. The whole flow microreactor system was dipped in a water bath (24 °C). A solution of 2,2'-dibromobiphenyl (0.10 M) in THF (flow rate = 6.00 mL min^−1^) and a solution of BuLi (0.50 M) in hexane (flow rate = 1.20 mL min^−1^) were introduced to M1 (*φ* = 250 μm). The resulting solution was passed through R1 (*φ* = 500 μm, *l* = 3.5 cm) and was mixed with a solution of xanthone **4X** (0.20 M) in THF (flow rate = 3.00 mL min^−1^) in M2 (*φ* = 500 μm). The resulting solution was passed through R2 (*φ* = 1000 μm, *l* = 50 cm) and was introduced to M3 (*φ* = 500 μm) where the solution was mixed with a solution of BuLi (0.50 M) in hexane (flow rate = 1.44 mL min^−1^). The resulting solution was passed through R3 (*φ* = 1000 μm, *l* = 200 cm) and was introduced to M4 (*φ* = 500 μm) where the solution was mixed with a solution of 4,4'-bis(dimethylamino)benzophenone **4N** (0.10 M) in THF (flow rate = 7.20 mL min^−1^). The resulting solution was passed through R4 (*φ* = 1000 μm, *l* = 200 cm). After a steady state was reached, the product solution was collected for 60 s and was treated with BuLi (1.67 M) in hexane (2.0 mL) to consume excess ketones and quench the reaction with water. After diluted with H_2_O, the whole mixture was extracted with Et_2_O. The combined organic layers were washed with water and brine, and dried over anhydrous Na_2_SO_4_. After filtration, solvent was concentrated under reduced pressure. The residue was purified by column chromatography on silica gel (hexane/EtOAc = 5) to give **3NX** (227 mg) as a colorless solid in 61% yield.

Mp 143–152 °C (decomp.); ^1^H NMR (400 MHz, CDCl_3_, 24 °C) *δ*/ppm 7.63 (1H, dd, J = 7.8, 1.5 Hz), 7.39 (1H, dd, J = 7.8, 1.5 Hz), 7.32 (1H, ddd, J = 7.8, 7.8, 1.5 Hz), 7.09–7.24 (6H, m), 7.02 (2H, AA’XX’), 6.95–7.00 (2H, m), 6.87 (2H, AA’XX’), 6.83–6.93 (3H, m), 6.72 (1H, ddd, J = 7.8, 7.8, 1.5 Hz), 6.64 (2H, AA’XX’), 6.59 (2H, AA’XX’), 6.18 (1H, dd, J = 7.8, 1.5 Hz), 5.72 (1H, m), 3.02 (1H, s), 2.94 (6H, s), 2.93 (6H, s); IR (KBr) 3048, 2796, 1612, 1520, 1478, 1450, 1354, 1240, 1190, 1154, 1032, 948, 816, 758 cm^−1^; FD-MS *m/z* 618 (M^+^, 100); Anal. Calcd. for C_42_H_38_N_2_O_3_: C, 81.53; H, 6.19; N, 4.53. Found: C, 81.60; H, 6.19; N, 4.40. 

### 4.9. Preparation of 9-[2'-Bis(4-Dimethylaminophenyl)Hydoroxymethylbiphenyl-2-yl]-9-Hydroxyxanthene **3NX** via Macro Batch Method

To a colorless solution of 2,2'-diiodobiphenyl (2.04 g, 5.02 mmol) in THF (20 mL) was added dropwise BuLi (1.47 mol dm^−3^ in hexane, 7.10 mL, 10.4 mmol) at 0 °C under Ar, and the mixture was stirred for 10 min at this temperature. To the resultant suspension of 2,2'-dilithiobiphenyl was added a mixture of 4,4'-bis(dimethylamino)benzophenone **4N** (1.30 g, 4.85 mmol) and xanthone **4X** (971 mg, 4.95 mmol) in THF (90 mL). After stirring for 2h at rt, the reaction was quenched by adding water. THF was evaporated, and the residue was extracted with benzene. The organic layer was washed with water and brine, and dried over Na_2_SO_4_. Evaporation of the solvent gave 3.66 g of oily material containing unsymmetric diol **3NX**. Two symmetric diols, **3NN** and **3XX**, were also formed in this reaction. Chromatographic separation on SiO_2_ (AcOEt/hexane, 1/4–1/2) followed by crystallization from MeOH gave **3NX** as colorless crystals (250 mg, y. 8.6%).

### 4.10. Preparation of 2-{Bis[3,4-Bis(Octyloxy)Phenyl]Hydroxymethyl}-2'-[Bis(4'-Methoxylphenyl)Hydroxymethyl]Biphenyl **3OO_8_**

An integrated flow microreactor system consisting of four T-shaped micromixers (M1, M2, M3, and M4), four microtube reactors (R1, R2, R3, and R4), and five microtube units [P1 (inner diameter *φ* = 1000 μm, length *l* = 100 cm), P2 (*φ* = 1000 μm, *l* = 50 cm), P3 (*φ* = 1000 μm, *l* = 100 cm), P4 (*φ* = 1000 μm, *l* = 50 cm), and P5 (*φ* = 1000 μm, *l* = 100 cm)] was used. The whole flow microreactor system was dipped in a water bath (24 °C). A solution of 2,2'-dibromobiphenyl (0.10 M) in THF (flow rate = 6.00 mL min^−1^) and a solution of BuLi (0.50 M) in hexane (flow rate = 1.20 mL min^−1^) were introduced to M1 (*φ* = 250 μm). The resulting solution was passed through R1 (*φ* = 500 μm, *l* = 3.5 cm) and was mixed with a solution of 4,4'-dimethoxybenzophenone **4O** (0.20 M) in THF (flow rate = 3.00 mL min^−1^) in M2 (*φ* = 500 μm). The resulting solution was passed through R2 (*φ* = 1000 μm, *l* = 50 cm) and was introduced to M3 (*φ* = 500 μm) where the solution was mixed with a solution of BuLi (0.50 M) in hexane (flow rate = 1.44 mL min^−1^). The resulting solution was passed through R3 (*φ* = 1000 μm, *l* = 200 cm) and was introduced to M4 (*φ* = 500 μm) where the solution was mixed with a solution of 3,3',4,4'-tetrakis(octyloxy)benzophenone **4O_8_** (0.10 M) in THF (flow rate = 7.20 mL min^−1^). The resulting solution was passed through R4 (*φ* = 1000 μm, *l* = 200 cm). After a steady state was reached, the product solution was collected for 120 s and was treated with H_2_O to quench the reaction.

After diluted with H_2_O, the whole mixture was extracted with CH_2_Cl_2_. The combined organic layers were washed with water and brine, and dried over anhydrous Na_2_SO_4_. After filtration, solvent was concentrated under reduced pressure. The residue was purified by column chromatography on silica gel (CH_2_Cl_2_/hexane = 3) to give **3OO_8_** (889 mg) as a colorless oil in 68% yield.

^1^H NMR (300 MHz, CDCl_3_) *δ*/ppm 7.11–7.00 (6H, m), 6.85–6.72 (12H, m), 6.62 (1H, dd, *J* = 8.4, 1.8 Hz), 6.52 (1H, dd, *J* = 8.4, 1.8 Hz), 6.14–6.08 (2H, m), 4.39 (1H, s), 4.19 (1H,s), 4.00 (2H, t, *J* = 6.6 Hz), 3.97 (2H, t, *J* = 6.6 Hz), 3.90–3.74 (10H, m), 1.89–1.63 (8H, m), 1.53–1.18 (40H, m), 0.94–0.82 (12H, m); ^13^C NMR (100 MHz, CDCl_3_) *δ*/ppm 158.53, 158.45, 148.35, 148.31, 148.26, 147.75, 143.94, 143.87, 141.12, 140.88, 138.98, 138.80, 131.58, 131.33, 130.31, 130.18, 129.64, 128.53, 125.98, 125.93, 125.79, 121.16, 120.34, 115.04, 113.10, 112.97, 112.87, 112.57, 83.35, 83.17, 69.27, 69.20, 69.12, 68.95, 55.18, 55.15, 31.78, 29.41, 29.35, 29.23, 29.13, 25.98, 25.94, 22.62, 14.05; IR (neat) 3538, 3397, 3053, 2953, 2927, 2856, 1607, 1583, 1509, 1468, 1442, 1435, 1417, 1379, 1298, 1252, 1175, 1162, 1137, 1064, 1037, 1064, 1037, 828, 808, 789, 756 cm^−1^; FD-MS *m*/*z* 1091 (M^+^, 83), 1090 (BP); FD-HR-MS (FD) Calcd. for C_72_H_98_O_8_: 1090.7262 Found: 1090.7244.

### 4.11. Preparation of 2-{Bis[3,4-Bis(Hexadecyloxy)Phenyl]Hydroxymethyl}-2'-[Bis(4'-Methoxylphenyl)Hydroxymethyl]Biphenyl **3OO_16_**

An integrated flow microreactor system consisting of four T-shaped micromixers (M1, M2, M3, and M4), four microtube reactors (R1, R2, R3, and R4), and five microtube units [P1 (inner diameter *φ* = 1000 μm, length *l* = 100 cm), P2 (*φ* = 1000 μm, *l* = 50 cm), P3 (*φ* = 1000 μm, *l* = 100 cm), P4 (*φ* = 1000 μm, *l* = 50 cm), and P5 (*φ* = 1000 μm, *l* = 100 cm)] was used. The whole flow microreactor system was dipped in a water bath (24 °C). A solution of 2,2'-dibromobiphenyl (0.10 M) in THF (flow rate = 6.00 mL min^−1^) and a solution of BuLi (0.50 M) in hexane (flow rate = 1.20 mL min^−1^) were introduced to M1 (*φ* = 250 μm). The resulting solution was passed through R1 (*φ* = 500 μm, *l* = 3.5 cm) and was mixed with a solution of 4,4'-dimethoxybenzophenone **4O** (0.20 M) in THF (flow rate = 3.00 mL min^−1^) in M2 (*φ* = 500 μm). The resulting solution was passed through R2 (*φ* = 1000 μm, *l* = 50 cm) and was introduced to M3 (*φ* = 500 μm) where the solution was mixed with a solution of BuLi (0.50 M) in hexane (flow rate = 1.44 mL min^−1^). The resulting solution was passed through R3 (*φ* = 1000 μm, *l* = 200 cm) and was introduced to M4 (*φ* = 500 μm) where the solution was mixed with a solution of 3,3',4,4'-tetrakis(hexadecyloxy)benzophenone **4O_16_** (0.10 M) in toluene (flow rate = 7.20 mL min^−1^). The resulting solution was passed through R4 (*φ* = 1000 μm, *l* = 200 cm). After a steady state was reached, the product solution was collected for 60 s and was treated with H_2_O to quench the reaction. 

After diluted with H_2_O, the whole mixture was extracted with CH_2_Cl_2_. The combined organic layers were washed with water and brine, and dried over anhydrous Na_2_SO_4_. After filtration, solvent was concentrated under reduced pressure. The residue was purified by column chromatography on silica gel (CH_2_Cl_2_/hexane = 2) and GPC separation (1+2H) to give **3OO_16_** (889 mg) as a colorless oil in 68% yield, which was solidified upon standing.

Mp 60–62 °C; ^1^H NMR (300 MHz, CDCl_3_) *δ*/ppm 7.11–6.99 (6H, m), 6.85–6.72 (12H, m), 6.63 (1H, dd, *J* = 8.4, 1.8 Hz), 6.52 (1H, dd, *J* = 8.4, 1.8 Hz), 6.14–6.08 (2H, m), 4.39 (1H, s), 4.19 (1H,s), 4.00 (2H, t, *J* = 6.6 Hz), 3.96 (2H, t, *J* = 6.6 Hz), 3.90–3.73 (10H, m), 1.89–1.62 (8H, m), 1.52–1.18 (104H, m), 0.92-0.82 (12H, m); ^13^C NMR (100 MHz, CDCl_3_) *δ*/ppm 158.56, 158.50, 148.40, 148.36, 148.28, 147.79, 143.97, 143.90, 141.16, 140.91, 140.65, 139.02, 138.85, 131.61, 131.36, 130.34, 130.21, 129.67, 128.57, 126.02, 125.97, 125.85, 121.20, 120.37, 115.11, 113.15, 113.00, 112.92, 112.62, 83.38, 83.20, 69.32, 69.25, 69.17, 69.02, 55.23, 55.20, 31.92, 29.71, 29.66, 29.51, 29.46, 29.37, 29.30, 29.20, 26.06, 26.02, 25.99, 22.68, 14.10; IR (KBr) 3382, 3051, 2919, 2850, 1607, 1510, 1468, 1434, 1416, 1389, 1302, 1254, 1173, 1138, 1064, 1039, 827, 810, 760, 721, 560 cm^−1^; FD-MS *m*/*z* 1540 (BP), 1539 (M^+^, 82); FD-HR-MS (FD) Calcd. for C_104_H_162_O_8_: 1539.2270 Found: 1539.2297.

### 4.12. Preparation of 3,3',4,4'-Tetrakis(Octyloxy)Benzophenone **4O_8_**

To a solution of 4-bromo-1,2-bis(octyloxy)benzene (4.14 g, 10.0 mmol) in 50 mL of dry ether was added BuLi in hexane(1.57 M, 6.40 mL, 10.0 mmol) at 22 °C under Ar, and the mixture was stirred for 1 h. To the suspension was added *N*-carboethoxypiperidine (770 µL, 4.99 mmol) and the mixture was stirred at 23 °C for 20 h. After diluted with 3 M HCl aq., the whole mixture was extracted with CH_2_Cl_2_. The combined organic layers were washed with 3 M HCl aq. and 1 M NaOH aq., and dried over anhydrous MgSO_4_. After filtration, solvent was concentrated under reduced pressure. The residue was purified by column chromatography on silica gel (CH_2_Cl_2_/hexane = 2) to give **4O_8_** (2.92 g) as a colorless solid in 84% yield.

Mp 58.5–59.5°C; ^1^H NMR (300 MHz, CDCl_3_) *δ*/ppm 7.40 (2H, d, *J* = 1.8 Hz), 7.35 (2H, dd, *J* = 8.4, 1.8 Hz), 6.88 (2H, d, *J* = 8.4 Hz), 4.07 (4H, t, *J* = 6.6 Hz), 4.04 (4H, t, *J* = 6.6 Hz), 1.93–1.77 (8H, m), 1.55–1.21 (40H, m), 0.93–0.82 (12H, m); ^13^C NMR (100 MHz, CDCl_3_) *δ*/ppm 194.63, 152.74, 148.64, 130.69, 124.67, 114.65, 111.50, 69.28, 69.07, 31.82, 29.35, 29.27, 29.20, 29.10, 25.99, 22.67, 14.10; IR (KBr) 2955, 2920, 2873, 2852, 1665, 1597, 1581, 1518, 1469, 1413, 1390, 1343, 1301, 1265, 1237, 1200, 1127, 1023, 1011, 964, 847, 821, 753, 721, 626 cm^−1^; FD-MS *m*/*z* 695 (M^+^, BP); Anal. Calcd. for C_45_H_74_O_5_: C, 77.76; H, 10.73. Found: C, 77.76; H, 10.71%.

### 4.13. Preparation of 3,3',4,4'-Tetrakis(Hexadecyloxy)Benzophenone **4O_16_**

To a solution of 4-bromo-1,2-bis(hexadecyloxy)benzene (5.01 g, 7.85 mmol) in 25 mL of dry ether and 25 mL of dry hexane was added BuLi in hexane (1.57 M, 5.00 mL, 7.85 mmol) at 23 °C under Ar, and the mixture was stirred for 1 h. To the suspension was added *N*-carboethoxypiperidine (600 µL, 3.89 mmol) and the mixture was stirred at 24 °C for 23 h. After diluted with 3 M HCl aq., the whole mixture was extracted with CH_2_Cl_2_. The combined organic layers were washed with 3 M HCl aq. and 1 M NaOH aq., and dried over anhydrous MgSO_4_. After filtration, solvent was concentrated under reduced pressure. The residue was purified by column chromatography on silica gel (CH_2_Cl_2_/hexane = 1) to give **4O_16_** (3.56 g) as a colorless solid in 80% yield.

Mp 81.5–83.0 °C; ^1^H NMR (300 MHz, CDCl_3_) *δ*/ppm 7.40 (2H, d, *J* = 1.8 Hz), 7.34 (2H, dd, *J* = 8.4, 1.8 Hz), 6.88 (2H, d, *J* = 8.4 Hz), 4.07 (4H, t, *J* = 6.6 Hz), 4.03 (4H, t, *J* = 6.6 Hz), 1.92–1.76 (8H, m), 1.53–1.18 (104H, m), 0.92–0.82 (12H, m); ^13^C NMR (100 MHz, CDCl_3_) *δ*/ppm 194.61, 152.74, 148.64, 130.69, 124.67, 114.65, 111.49, 69.28, 69.07, 31.93, 29.71, 29.64, 29.43, 29.37, 29.20, 29.12, 26.01, 22.68, 14.11; IR (KBr) 2956, 2917, 2849, 1661, 1597, 1579, 1518, 1468, 1432, 1414, 1392, 1342, 1266, 1237, 1200, 1125, 1030, 1016, 850, 754, 721, 625 cm^−1^; FD-MS *m*/*z* 1143 (M^+^, BP); Anal. Calcd. for C_77_H_138_O_5_: C, 80.85; H, 12.16. Found: C, 80.73; H, 12.15%.

### 4.14. Preparation of 4-Bromo-1,2-Bis(Hexadecyloxy)Benzene

To a solution of 1,2-bis(hexadecyloxy)benzene (14.9 g, 26.7 mmol) in 200 mL of dry CH_2_Cl_2_ was added *N*-bromosuccinimide (4.75 g, 26.7 mmol) in dry MeCN 70 mL at 23 °C under Ar, and the mixture was stirred for 20 h. After diluted with water, the whole mixture was extracted with CH_2_Cl_2_. The combined organic layers were washed with 10% Na_2_S_2_O_3_ aq. and brine, and dried over anhydrous MgSO_4_. After filtration, solvent was concentrated under reduced pressure. The residue was purified by column chromatography on silica gel (hexane/EtOAc = 200 to 100) to give 4-bromo-1,2-bis(hexadecyloxy)benzene (16.8 g) as a colorless solid in 99% yield.

Mp 62.5–63.5 °C; ^1^H NMR (300 MHz, CDCl_3_) *δ*/ppm 7.00–6.94 (2H, m), 6.72 (1H, d, *J* = 9.1 Hz), 3.95 (2H, t, *J* = 6.6 Hz), 3.94 (2H, t, *J* = 6.6 Hz), 1.86–1.72 (4H, m), 1.52–1.17 (52H, m), 0.92–0.84 (6H, m); ^13^C NMR (100 MHz, CDCl_3_) *δ*/ppm 150.06, 148.38, 123.42, 116.95, 115.17, 112.79, 69.53, 69.38, 31.93, 29.71, 29.63, 29.38, 29.23, 29.13, 25.99, 22.70, 14.11; IR (KBr) 2917, 2848, 1588, 1509, 1503, 1468, 1446, 1434, 1392, 1324, 1258, 1220, 1132, 1066, 1014, 955, 865, 842, 826, 800, 722, 644, 576 cm^−1^; FD-MS *m*/*z* (relative intensity) 638 (M^+^, 35), 636 (M^+^, 35), 559 (BP); Anal. Calcd. for C_38_H_69_O_2_Br: C, 71.55; H, 10.90. Found: C, 71.37; H, 10.80%.

### 4.15. Measurement of Redox Potentials

All the redox potentials (*E*^ox^ and *E*^red^) were measured under argon atmosphere by cyclic voltammetry in CH_2_Cl_2_ containing 0.1 M Bu_4_NBF_4_ as a supporting electrolyte. All the values are reported in *E*/V *vs.* SCE, and Pt wire was used as the working electrode. In the case of irreversible waves, half-wave potentials were estimated from the peak potentials as *E*^ox^ = *E*^pa^ (anodic peak potential) −0.03 V, and *E*^red^ = *E*^pc^ (cathodic peak potential) +0.03 V.

### 4.16. X-Ray Structural Analyses

Unsymmetric DHP **1NX**: C_42_H_36_N_2_O, *M* 584.76, 0.60 × 0.10 × 0.05 mm, orthorhombic, space group *P*2_1_2_1_2_1_, *a* = 10.4511(4), *b* = 14.0601(7), *c* = 20.771(1) Å, *V* = 3052.2(3) Å [3,4], and *D*_calcd_ (Z = 4) = 1.272 g cm^−3^. Numerical absorption correction was applied (μ = 0.76 cm^−1^). Data collection was performed on a Rigaku Mercury CCD camera appararus (Mo-Kα radiation, λ = 0.71069 Å, 50 kV, 50 mA) at 203 K. The structure was solved by the direct method and refined by full-matrix least-squares method on *F* with anisotropic temperature factors for non-hydrogen atoms. Hydrogen atoms were located at the calculated positions. The final *R* and *Rw* values are 0.044 and 0.049 for 407 parameters and 2835 reflections with *I* > 3σ (*I*) (independent reflections, 3625; 2θ_max_ = 53.5°). The GOF indicator is 1.20. Residual electron density is 0.16 Å^−3^. CCDC 182/1003
